# ^18^F-Fluorodeoxyglucose positron emission tomography/computed tomography findings in a patient with bilateral macronodular adrenal hyperplasia

**DOI:** 10.1259/bjrcr.20200034

**Published:** 2020-05-11

**Authors:** Bo Pan, Shicun Wang, Zongke Chen, Guichang Zou

**Affiliations:** 1 PET/CT Center, The First Affiliated Hospital of University of Science and Technology of China, Hefei 23001, China; 2 Institute on Aging and Brain Disorders, The First Affiliated Hospital of USTC, Division of Life Sciences and Medicine, University of Science and Technology of China, Hefei 230026, China

## Abstract

Adrenocorticotropic hormone-independent macronodular adrenal hyperplasia (AIMAH) is a rare bilateral adrenocorticotropic hormone (ACTH)-independent nodular adrenal hyperplastic disease. Most patients with AIMAH are usually asymptomatic and only a small percentage present with subclinical or apparent Cushing’s syndrome caused by excessive corticosteroid secretion. Herein, we reported the case of a 51-year-old female with bilateral macronodular adrenal hyperplasia with mild fluorodeoxyglucose uptake based on PET/CT imaging findings. Her symptoms resolved after surgical resection of the right adrenal gland.

## Introduction

Clinically, hypercortisolism is usually caused by an unexpected long-term increase in glucocorticoid hormones in tissue circulation. Hypercortisolism is classified into two main categories: adrenocorticotropic hormone (ACTH)-dependent hypercortisolism and ACTH-independent hypercortisolism. ACTH-dependent hypercortisolism accounts for 80–85% of cases, of which 80% were pituitary tumours and 20% were cases of ectopic ACTH syndrome.^1,2^ ACTH-independent hypercortisolism accounts for 15~20% of all patients and is mainly composed of adrenocortical adenoma, adrenal cortical adenocarcinoma, ACTH-independent macronodular adrenal hyperplasia (AIMAH) and primary pigmented nodular adrenocortical disease. The incidence of AIMAH with hypercortisolism is less than 2%.^3^ We reported a case of bilateral macronodular adrenal hyperplasia in a 51-year-old female with PET/CT findings.

## Case report

**Figure 1.**
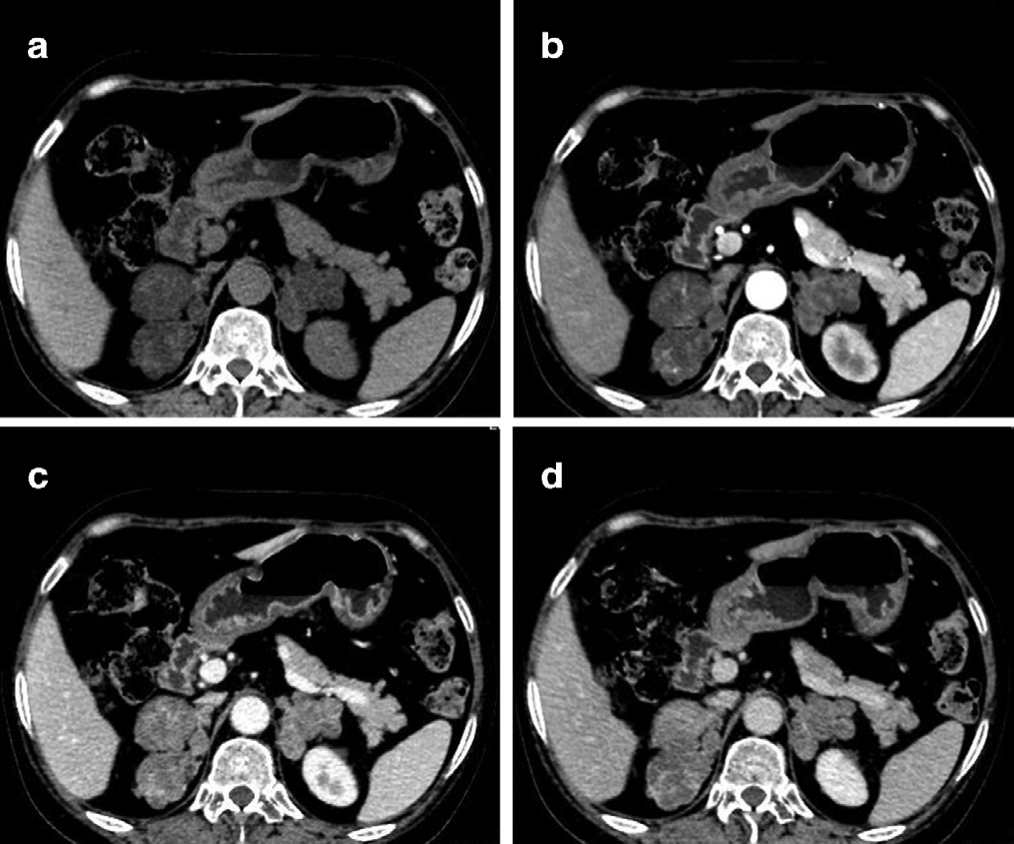
CT showing hypodense lobulated lesions on the right and left adrenal glands (A); Arterial phase images of enhanced CT revealing mild-to-moderate dotted and linear enhancement of the bilateral adrenal lesions (B); Bilateral adrenal lesions showing moderate enhancement in the portal phase (C); The degree of enhancement of the lesions in the venous phase decreased slightly (D) CT: computed tomography

**Figure 2.**
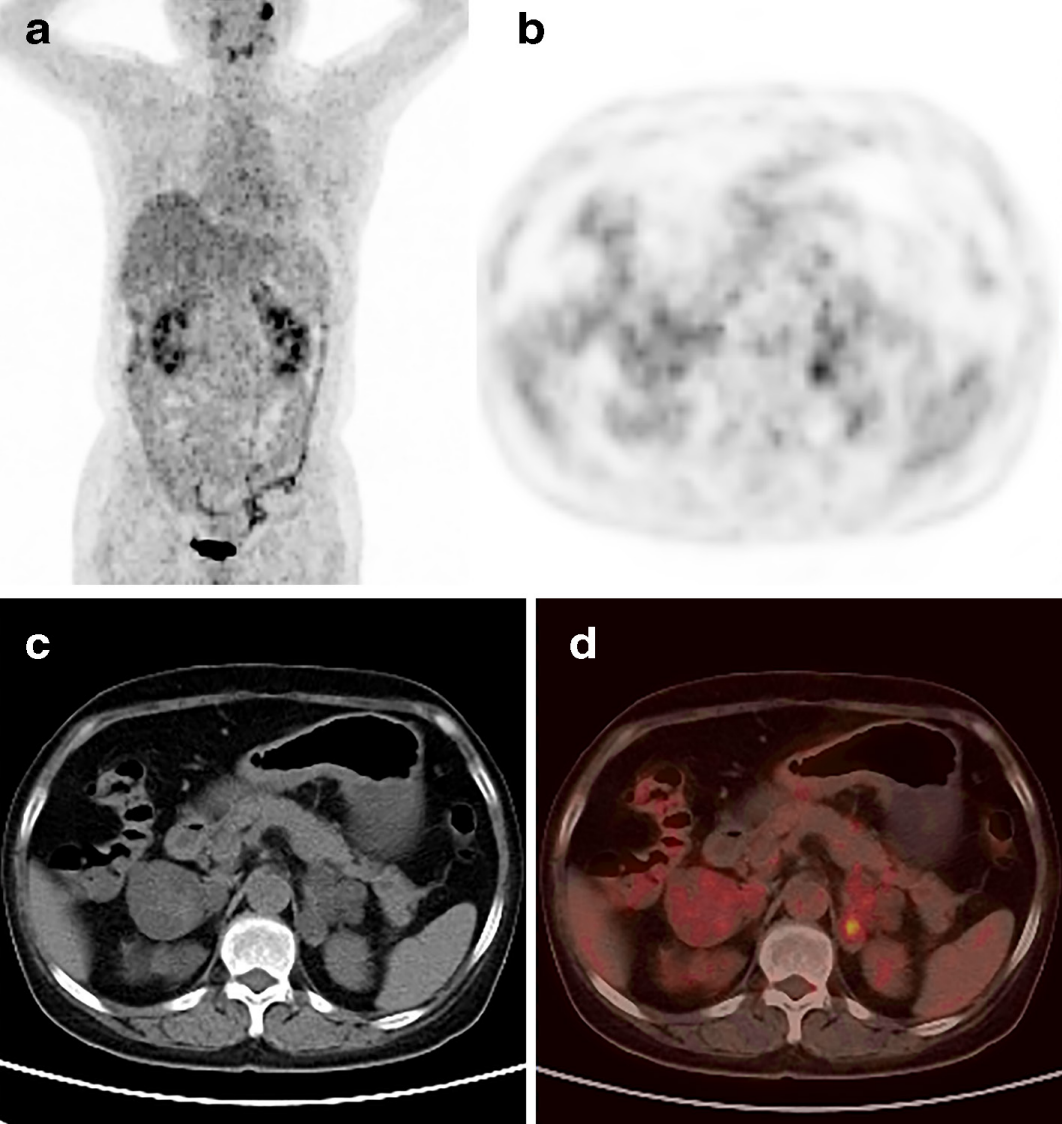
No obvious abnormality can be observed on the whole-body PET MIP image (A); Mild FDG uptake (SUVmax: 3.7) was observed in the right adrenal and left adrenal glands (SUVmax: 3.9) on axial PET (B); A cystic hypodensity with well-defined boundary and ginger-like appearance was observed on the adrenal gland with a CT value of 0 ~ 4 HU on the adrenal gland and 8 ~ 15 HU on the right adrenal gland on transversal CT (C); Bilateral adrenal lesions showed a slight increase in uptake on the fusion PET/CT images (D) PET: positron emission tomography, CT: computed tomography, MIP: maximum intensity projection, SUVmax: maximum standardised uptake value, FDG: fluorodeoxyglucose

**Figure 3.**
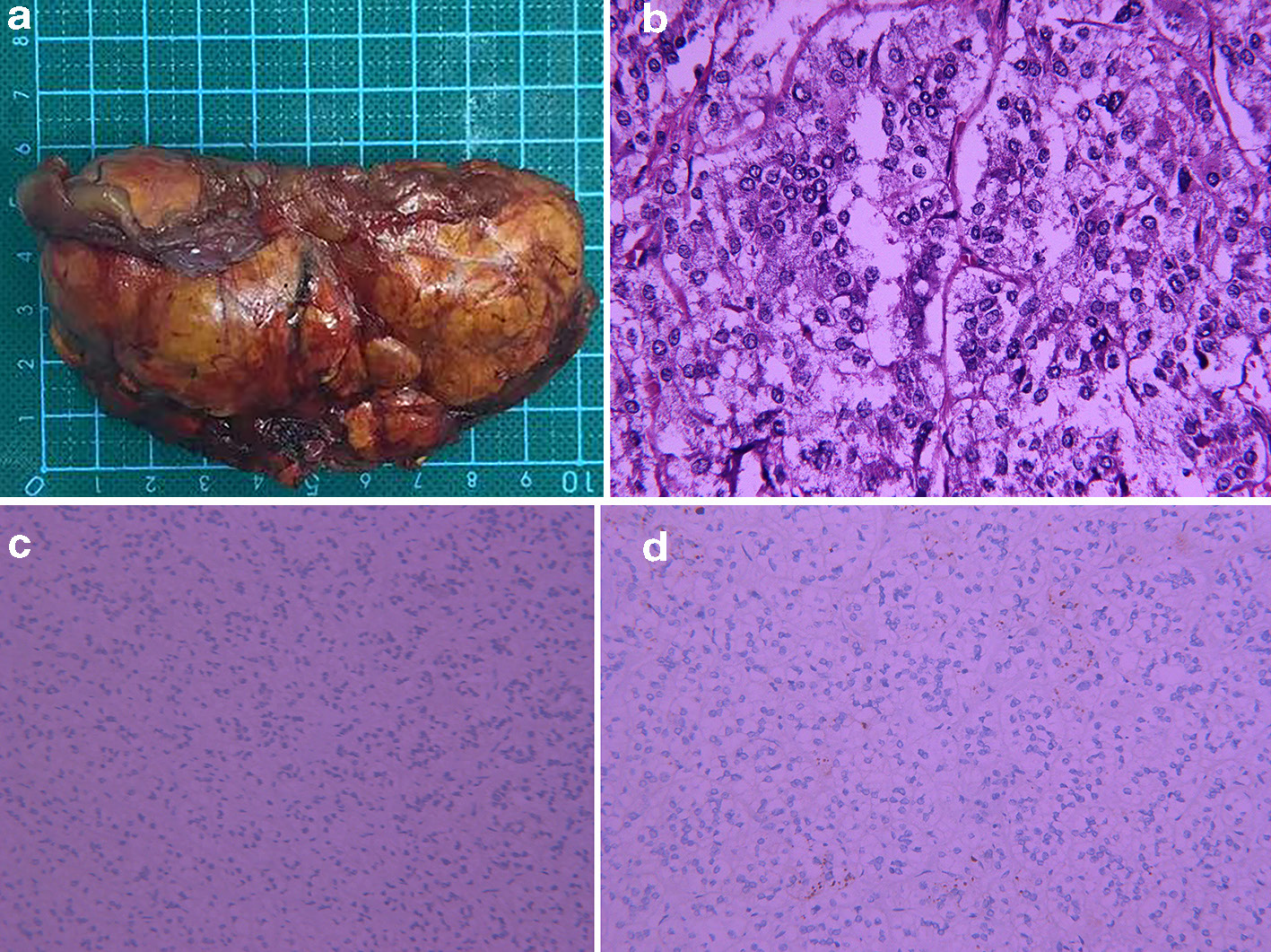
Gross pathological appearance of the right adrenal gland (A); Histopathology showed nodules of varying sizes under microscopy. The nodules were composed of compact cells and clear cells (haematoxylin and eosin, *B* × 200). Immunohistology for inhibin and melan A was weakly positive (approximately 10%) (C, D).

A 51-year-old female presented with oedema of the face and both lower extremities since more than 3 months. She had a history of hypertension for 30 years and cerebral haemorrhage 6 months ago, for which she received long-term standardised pharmacotherapy. Physical examination revealed typical Cushing’s syndrome appearance including moon facies and hairy and thin skin throughout the whole body. Serum aldosterone (690.90 pmol l^−1^) and 24 h urinary cortisol (2070.00 nmoL) were elevated. Laboratory tests also confirmed mild hypokalaemia and abnormal circadian rhythm of cortisol concentration. No obvious abnormality was observed on pituitary MRI. CT revealed lobulated hypodense masses in both adrenal glands (Figure 1), following which a diagnosis of bilateral adrenal metastases was made. ^18^F-fluorodeoxyglucose (FDG) PET/CT examination with SIEMENS Biography 16 PET/CT scanner was performed to locate the primary tumour, PET images were acquired with two minutes per bed. The CT-based attenuation-corrected PET images were reconstructed with an iterative true X algorithm (true X 3D, three iterations, 24 subsets) and smoothed with a Gaussian filter with 4 mm FWHM (full width half maximum) (Matrix 168×168), which revealed mild FDG uptake in the adrenal lesions bilaterally (Figure 2). The patient’s immediate family also underwent bilateral adrenal CT examination to rule out familial genetic disease. Her father and sister underwent CT, which did not reveal any abnormality in the adrenal glands. The patient’s younger brother and son also underwent CT for the adrenal glands, and revealed bilateral adrenal lesions. She was not willing to undergo genetic testing, and a diagnosis of familial genetic AIMAH with Cushing’s syndrome was made on the basis of the imaging findings and family history. Right laparoscopic adrenalectomy was performed under general anaesthesia, and pathological examination suggested macronodular hyperplasia of the right adrenal gland (Figure 3). The patient was discharged 1 week after surgery. Her clinical symptoms disappeared, blood hormone levels returned to normal, and she appeared healthy during the 7-month follow-up.

## Discussion

The incidence of bilateral hyperplastic adrenal macronodules is very low. The literature is limited to case reports and no longitudinal studies with a large study population.^4^ Few reports on AIMAH have described the ^18^F-FDG PET/CT imaging findings, which revealed abnormally high uptakes of ^18^F-FDG.^5^ This condition can easily be misdiagnosed as metastasis or lymphoma. Most metastases have primary malignant lesions in another part of the body and most primary adrenal lymphomas are diffuse large B cell lymphomas with hypermetabolism. However, our case manifested mild FDG uptake on PET/CT image, the maximum standardized uptake value (SUVmax:3.9) is similar to the value of liver, which is different from the previous literature reported. The possible cause is that AIMAH is pathologically characterized by hyperplasia of cortical nodules and is not a real tumour, so its uptake value is similar to that of normal adrenal gland tissue, which showed a slight FDG uptake. Further studies are required based on large sample for mechanism of varied degree FDG uptake. Ginger-like appearance and retention of adrenal contour are the typical imaging features.^6^ The exact aetiology of bilateral adrenal macronodules is unclear and genetic variance in ARMC5 may be the main aetiology.^7–9^ Accordingly, genetic testing is necessary if AIMAH is suspected. AIMAH commonly causes Cushing’s syndrome. Several patients undergo unilateral adrenalectomy of the larger adrenal gland, which can help prevent the need for lifelong steroid replacement. Although unilateral adrenalectomy is safe and effective, subclinical cortisol hyperplasia may occur after the procedure. Therefore, contralateral adrenalectomy was necessary. Thus, close follow-up is essential for patients who have undergone unilateral adrenalectomy. One-stage bilateral adrenalectomy may be the treatment of choice for elderly patients.^10–12^

A diagnosis of non-ACTH-dependent hypercortisolism should be considered if both adrenal glands show ginger-like appearance on CT and a slight FDG uptake on PET/CT, without an increase in FDG uptake in other parts of the body. The final diagnosis of AIMAH depends on postoperative histopathology.

## Learning points

1.The typical characteristics of adrenocorticotropic hormone-independent macronodular adrenal hyperplasia (AIMAH) include ginger-like appearance on CT image, retention of basic adrenal morphology, and mild-to-moderate fluorodeoxyglucose (FDG) uptake. Meanwhile, the other parts of the whole-body PET imaging are negative.

2.^18^F-FDG PET/CT imaging could distinguish primary adrenal lymphoma and metastatic adrenal cancer from AIMAH, and offers the evidence for later surgical resection.
